# The Balancing Act in Ferroelectric Transistors: How Hard Can It Be?

**DOI:** 10.3390/mi9110582

**Published:** 2018-11-07

**Authors:** Raymond J. E. Hueting

**Affiliations:** MESA+ Institute for Nanotechnology, University of Twente, P.O. Box 217, 7500AE Enschede, The Netherlands; r.j.e.hueting@utwente.nl

**Keywords:** CMOS, field-effect transistor, ferroelectrics, MOS devices, negative-capacitance, piezoelectrics, power consumption

## Abstract

For some years now, the ever continuing dimensional scaling has no longer been considered to be sufficient for the realization of advanced CMOS devices. Alternative approaches, such as employing new materials and introducing new device architectures, appear to be the way to go forward. A currently hot approach is to employ ferroelectric materials for obtaining a positive feedback in the gate control of a switch. This work elaborates on two device architectures based on this approach: the negative-capacitance and the piezoelectric field-effect transistor, i.e., the NC-FET (negative-capacitance field-effect transistor), respectively π-FET. It briefly describes their operation principle and compares those based on earlier reports. For optimal performance, the adopted ferroelectric material in the NC-FET should have a relatively wide polarization-field loop (i.e., “hard” ferroelectric material). Its optimal remnant polarization depends on the NC-FET architecture, although there is some consensus in having a low value for that (e.g., HZO (Hafnium-Zirconate)). π-FET is the piezoelectric coefficient, hence its polarization-field loop should be as high as possible (e.g., PZT (lead-zirconate-titanate)). In summary, literature reports indicate that the NC-FET shows better performance in terms of subthreshold swing and on-current. However, since its operation principle is based on a relatively large change in polarization the maximum speed, unlike in a π-FET, forms a big issue. Therefore, for future low-power CMOS, a hybrid solution is proposed comprising both device architectures on a chip where hard ferroelectric materials with a high piezocoefficient are used.

## 1. Introduction

As is commonly known, the key component of the microprocessor, the conventional metal-oxide-semiconductor field-effect transistor (MOSFET), needs some refurbishment. The traditional dimensional scaling of this device as proposed earlier [1] no longer suffices to cope with present day requirements. Other device architectures, such as FinFETs [2,3] and ultrathin-body (UTB) devices [4,5], and integration of other materials, such as silicon-germanium (e.g., [6]), are presently in production.

However, despite these adjustments, for several years, the maximum supply voltage of the microprocessor has been around 0.7–0.8 V. The main reason for this is to limit the static (or off-state) power consumption that is governed by the off-state current ($I_{\mathrm{OFF}}$) [7], and that rises exponentially for a reduced threshold voltage. The latter is because the current below the threshold voltage, i.e., the subthreshold current, is a diffusion or thermionic emission current. Its slope against the gate-source voltage ($V_{\mathrm{GS}}$) has a maximum value dictated by Boltzmann’s tyranny being ∼60 mV/dec at room temperature, or, equivalently, a minimum ideality factor *m* equal to unity.

To break this tyranny, alternative device architectures have been proposed based on other physical principles, such as tunnel FETs [8,9,10,11] and impact-ionization MOSFETs [12]. Although these architectures have a strong potential, the realization of those are relatively difficult.

**Figure 1:**
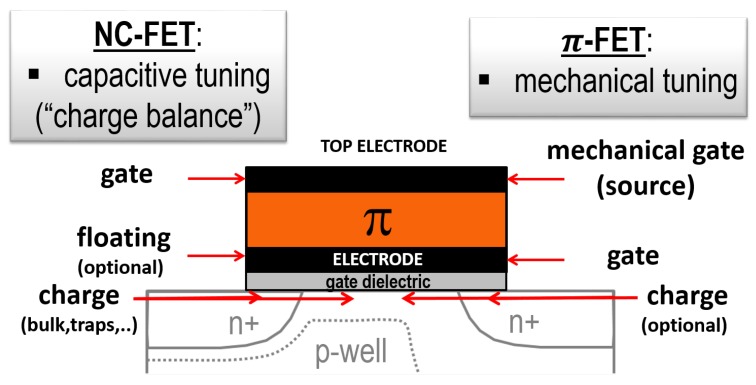
Schematic cross-section of a bulk NC-FET (left) and π-FET. Both device concepts comprise a ferroelectric material for obtaining a positive feedback in the gate control of the current in a conventional MOSFET. In case of the NC-FET, the top electrode represents the gate, and there is an optional floating electrode for technological reasons and to smear out potential fluctuations in the channel. The amount of charge (“charge balance”) in or near the body/channel is important here. For the π-FET, the top electrode is a rigid mechanical gate connected to the source, and there is a conventional gate. The electromechanical properties of the semiconductor and mechanical boundary conditions are important here. The figures are not at scale.

Later, various device architectures have been proposed in which a ferroelectric (FE) layer has been embedded in a conventional MOSFET (see Figure 1): the negative-capacitance field-effect transistor (NC-FET) [13,14], and the piezoelectric field-effect transistor (π-FET) [15,16]. In particular, the NC-FET is currently a hot topic since, in principle, it only requires a single additional FE layer that is compatible with CMOS technology. Although the charge transport physics in both device architectures is basically that of a conventional MOSFET, the physics involved inside the gate stack is different, as discussed in the following sections.

Before discussing the device architectures, first the physics and characteristics of FE materials should be briefly explained. For a more thorough overview, refer to [17,18].

**Figure 2:**
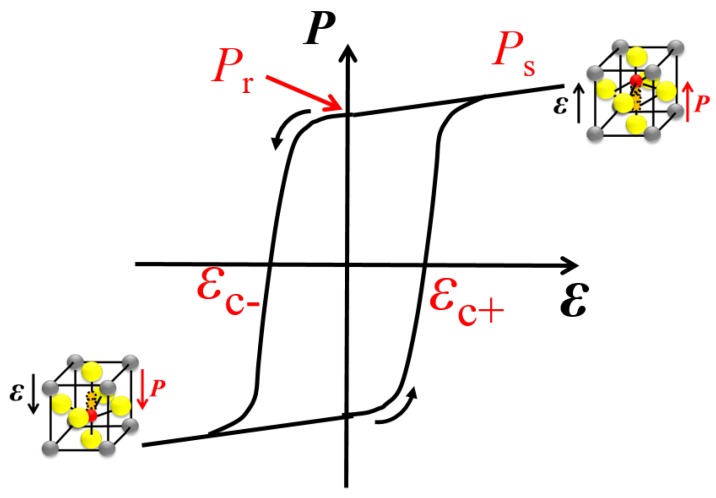
Schematic *P*-E curve illustrating the hysteretic effect caused by internal dipoles. The remnant polarization $P_{r}$ represents the polarization at zero field. The saturation polarization $P_{s}$ is the maximum polarization at which a spontaneously formed dipole moment has been formed. $E_{C-}$ and $E_{C+}$ are the coercive fields at which the hysteresis loop intersects the negative respectively positive field axis.

FE materials consist of fixed ions in preferably a perfect crystalline lattice. Depending on the asymmetry in the lattice those ions form dipoles, which in turn form domains depending on the quality of the material. Because of those dipoles, a hysteretic polarization-electric field (*P*-E) curve is obtained (see Figure 2). Important figures-of-merit (FOMs) in FE materials are the coercive electric field ($E_{C}$) or coercive voltage, the remnant polarization ($P_{r}$), and the saturation (or spontaneous) polarization ($P_{s}$). $E_{C}$ represents the strength of the applied field for which the polarization direction of (most of) the dipoles flips in the opposite direction. $P_{r}$ represents the resulting polarization value obtained when the applied varying (increasing or decreasing) electric field becomes zero (E = 0). Furthermore, since FE materials are piezoelectric (π-) materials (not vice versa), the π-coefficient ($d_{33}$) is also an important FOM in this context. This parameter in turn depends on $P_{s}$ [19,20]. The reason why for many sensor and transducer applications FE materials are used is because those materials, in particular perovskite ferroics (e.g., lead-zirconate-titanate, PZT), have a relatively high $P_{s}$ hence $d_{33}$ value. Note that, for most cases, the polarization can be simply be considered to be equal to the areal charge density (*Q*).

This work is outlined as follows. In Section 2 and Section 3, the basic operation principle of the NC-FET respectively π-FET is briefly explained. In Section 4, both devices are compared based on previous reports. Finally, in Section 5, the conclusions are drawn.

## 2. The Negative-Capacitance Field-Effect Transistor

**Figure 3:**
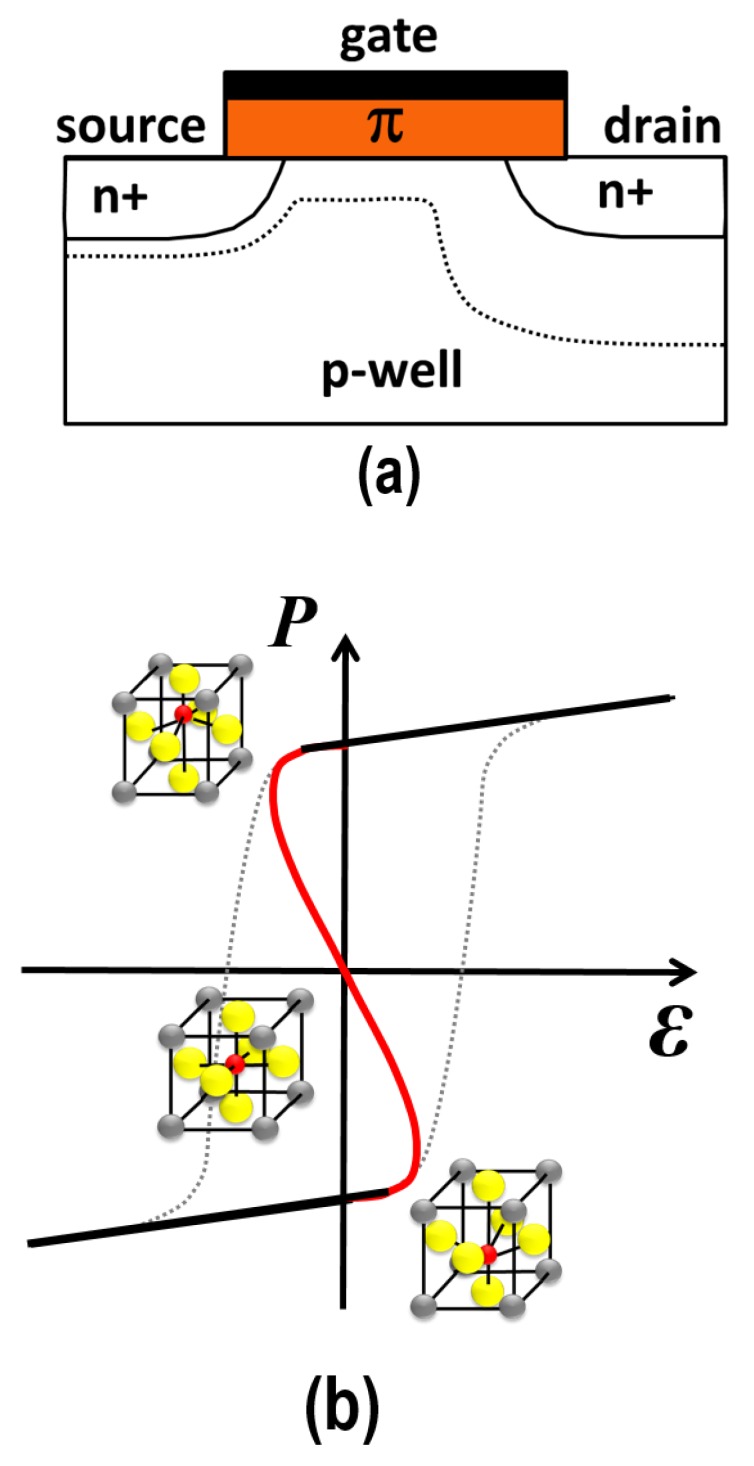
(**a**) schematic cross-section of the originally proposed NC-FET; (**b**) the “S”-shaped *P*-E curve highlighting the negative slope ∂P/∂E hence negative capacitance. The NC-FET operation is inside the *P*-E loop.

Recently, in the semiconductor community, a wide interest has formed for adopting the negative-capacitance (NC-) effect in reducing the power consumption of the FET, as originally proposed by Salahuddin and Datta [13,14]. Figure 3 shows a schematic cross-section of the NC-FET in which a poled FE layer is integrated, denoted π. The NC-effect originates from the hysteretic *P*-E loop that is present in ferroelectrics [17]. According to a proposed theory [21,22], during switching in field polarity, the ferroelectric polarization state does not follow the hysteretic loop. Instead, an internal S-shaped *P*-E curve is followed in which a part of its slope becomes negative. Hence, a negative permittivity, and thus a correspondingly negative capacitance are also obtained. This NC-effect is difficult to measure directly; it is energetically an unstable situation. However, by placing the FE layer on top of (or below) a conventional dielectric layer (e.g., SiO${}_{2}$ or Si) in a metal-insulator-metal (MIM) stack, the total capacitance of the stack increases, as experimentally shown for PZT/STO MIM capacitors [23]. (STO or SrTiO${}_{3}$ stands for strontium-titanate).

The drain current of a long channel MOSFET depends on the voltage division between the oxide or insulator capacitance ($C_{\pi }$) and the silicon capacitance ($C_{s}$), or to be more specific by the surface potential [24]:

(1) $$\psi_{s}=V_{\mathrm{GS}}\cdot \frac{C_{\mathrm{tot}}}{C_{s}}=V_{\mathrm{GS}}\cdot \frac{C_{\pi }}{C_{s}+C_{\pi }}=V_{\mathrm{GS}}\cdot \frac{1}{m},$$

with $C_{\mathrm{tot}}$ being the total capacitance of the gate-stack. If we now consider that $C_{\pi }$ is negative such that $C_{s}$ is just a little bit smaller than $C_{\pi }$, then there is voltage amplification ($\psi_{s}/V_{\mathrm{GS}}\gg 1$) and, therefore, the ideality factor *m* becomes less than one (m≪1) [13,23,25]. Consequently, a steep subthreshold swing (SS) can be obtained. In this way, a maximum $C_{\mathrm{tot}}$ is obtained excluding any undesired hysteresis effects.

Much research work followed studying the NC-effect (e.g., [26,27,28,29,30,31,32,33,34,35]). However, it has been debated that there is a limit to this NC-effect because of the use of multi-domain ferroelectrics [36]. Furthermore, in practice, this so-called “charge-balance” [37] is difficult to obtain partly because the actual NC value is not accurately known. For obtaining this charge-balance, an extensively combined experiment-modeling effort is required for finally obtaining an improved FET. In addition, interface traps in the gate stack could also affect this charge balance [38] which can be understood from Equation (1) in case $C_{\mathrm{tot}}$ also includes the interface trap capacitance. Note that in case of multiple interfacial layers in the gate stack $C_{s}$ in Equation (1) should be replaced by a capacitance representing the stack below the FE material (e.g., $C_{\mathrm{MOS}}$ [39]).

The operation of the NC-FET is inside the hysteretic *P*-E loop. This implies that in practice a relatively high $C_{\pi }=\partial P/\partial E$ is required and that strain values are relatively low. For an effective use, a relatively wide P−E loop is needed [40] such that the coercive voltage is higher than the supply voltage of the FET ($V_{C}>V_{\mathrm{DD}}$). However, the optimal $P_{r}$ depends on the device architecture. For instance, for long channel bulk MOSFETs, it was experimentally shown that a perovskite FE material such as PZT ($P_{r}\approx 40\;\mu$C/cm${}^{2}$) results in an SS of ∼38 mV/dec [34]. Conversely, in fully-depleted (FD) devices, such as a FinFET, a “hard” FE material such as Hafnium-Zirconate (HZO, $P_{r}\approx 3\;\mu$C/cm${}^{2}$)) was employed reaching an SS of ∼55 mV/dec [32].

For illustration purposes, Figure 3 shows a symmetric hysteretic *P*-E loop. Such a symmetry strongly depends on the internal charge distribution of the FE layer induced either by the workfunction difference between its incorporated top and bottom electrodes (or semiconductor body) or by its internal parasitic (e.g., fixed) charge distribution. For instance, provided that the workfunction of the top metal is higher than that of the semiconductor body (or bottom electrode), the *P*-E curve shifts to the right on the horizontal E-scale. This will cause an asymmetry in the *P*-E curve, not desired for the NC-FET since that will reduce the operating bias range. If there was no difference between the electrode workfunctions and no parasitic charge, then the *P*-E curve would be symmetric.

## 3. The Piezoelectric Field-Effect Transistor

**Figure 4:**
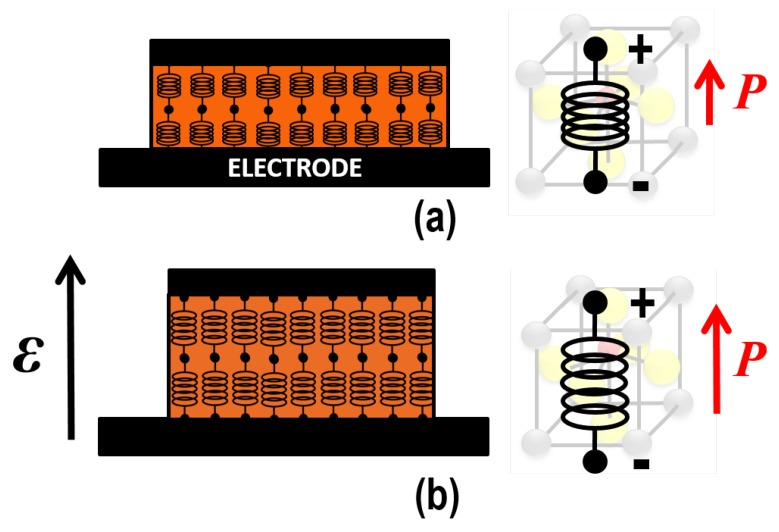
Illustration of the converse piezoelectric effect (**a**) at thermal equilibrium, and (**b**) when an external field has been applied. For illustration purposes, the change in thickness induced by this field has been exaggerated. In conventional piezoelectric materials, the polarization direction is fixed, while, in ferroelectric materials, the polarization depends on the electric field direction.

As stated before, FE materials are π-materials. In addition, generally, a π-material basically comprises dipoles because of the presence of fixed ions and asymmetry in the lattice. When stressing such material, there is some deformation, hence strain, which also results in a change in polarization, and therefore an electric field has formed. This is called the π-effect. The opposite is also possible, i.e., the converse π-effect [41]. When no mechanical stress (*T*) is applied to the π-material an external electric field (E) induces a deformational strain *S* according to (Figure 4):

(2) T=c·S+e·E,

with *c* being Young’s modulus and *e* being the π-charge constant.

In reality, all parameters in Equation (2) are tensors, but, for simplicity, we consider a one-dimensional system. Note that the π-coefficient $d_{33}=-e/c$, i.e., the amount of displacement or deformation in units length per unit applied voltage.

**Figure 5:**
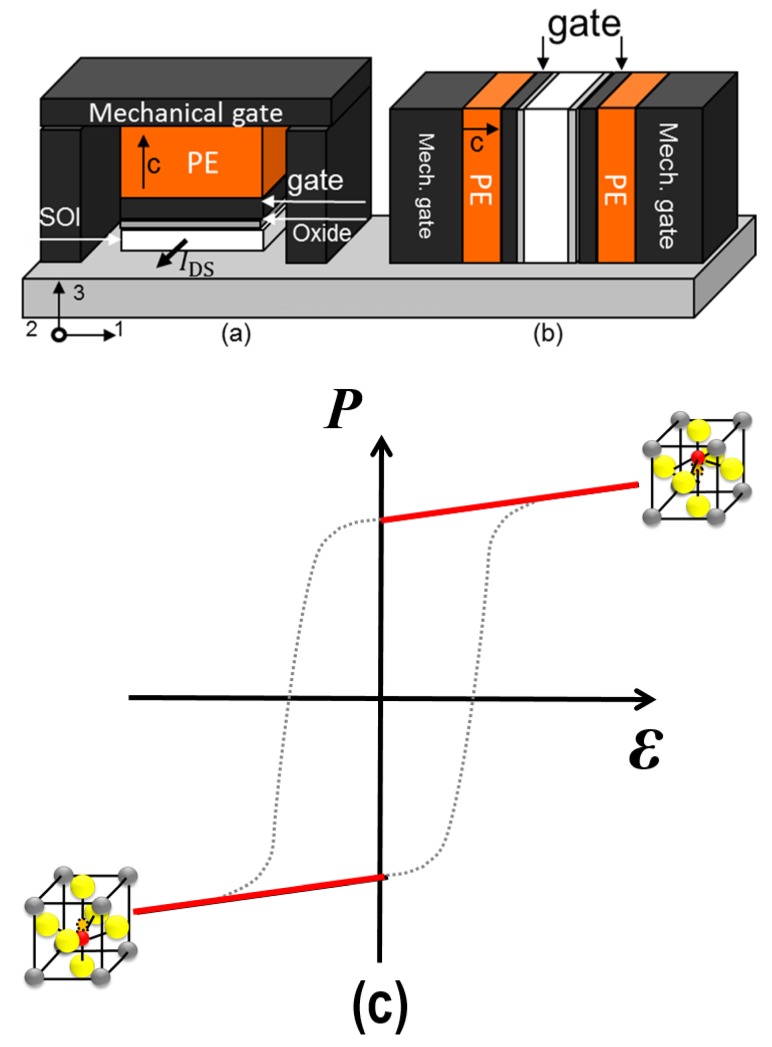
(**a**,**b**) schematic cross-section of the originally proposed π-FET (after [15]); (**c**) the highlighted operational regime of the π-FET which is outside the *P*-E loop.

More recently, we have proposed the so-called piezoelectric field-effect transistor (π-FET) [15,16] in which an FE layer is implemented (see Figure 5). The basic idea is that the strain in the semiconductor body can be tuned by the converse π-effect. As a result, during device operation, the semiconductor body is relaxed in the off-state, resulting in a low off-current ($I_{\mathrm{OFF}}$), and it is strained in the on-state, resulting in a high $I_{\mathrm{ON}}$. The strain influences the semiconductor transport properties: it reduces the band gap and depending on the device/crystal orientation it also increases the charge carrier mobility. This reduces the SS [16].

In our theoretical and numerical work [16,42], we have predicted that there are experimental challenges to reduce the SS effectively: (1) ultrathin and relatively stiff interfacial layers in between the π-layer and semiconductor body are required, all with proper (rigid) mechanical boundary conditions, (2) the semiconductor body should preferably have a low stiffness and a high “effective” deformation potential, and (3) the π-material should have a high π-response $d_{33}$ and a high electrical breakdown field.

Opposite to the NC-FET, the operation of the π-FET is outside the hysteretic *P*-E loop. This implies that a relatively low ∂P/∂E is required and that the strain values are relatively high. As stated before, for improving the converse π-effect in a FET, obviously a relatively high $d_{33}$ is needed. Since this parameter is proportional to $P_{s}$ [20], this implies that a relatively high *P*-E loop is required. In most cases, perovskite FE materials should be used here. PZT, for instance, is such a material, its $d_{33}$ (∼110 pm/V) is much higher than that of conventional π materials such as aluminium-nitride (AlN) or zinc-oxide (ZnO). Therefore, FE materials are commonly used for various sensor and transducer applications [18]. Hard materials such as HZO have a $d_{33}$ of ∼10 pm/V ($P_{r}\approx 8\;\mu$C/cm${}^{2}$) [43] comparable to that of conventional π-materials such as AlN.

From the view point of the device performance of the π-FET, the symmetry of the hysteretic *P*-E loop is less important than it is for the NC-FET, mainly because the overall height of the *P*-E curve is essential in this case.

Note that, around the same time, the piezoelectronic transistor (PET) was reported [44] in which the converse π-effect was employed to a highly pressure-sensitive piezoresistive material (e.g., Samarium-selenide, SmSe). Since the transport physics is not based on that of conventional MOSFETs, it has further not been considered in this comparative work. However, there are some interesting features that should be highlighted. First, the PET comprises a perovskite FE material (e.g., PZN-PT, PMN-PT or even PZT) and the mechanics in the PET was treated thoroughly. Second, rigid boundary conditions were used, and also geometry effects were considered. However, very importantly, the breakdown field of the FE material was ignored.

Earlier [16], a combination of technology computer aided design (TCAD) [45] and multiphysics FEM [46] tools was used to estimate the SS in germanium (Ge) FinFETs showing values of around 50 mV/dec. In order to achieve this targeted SS value 5 nm wide, 45 nm high, and 80 nm long fins were adopted. Table 1 summarizes some SS values for both n-type and p-type FETs in various bulk semiconductor materials. Later, other theoretical works [47] reported lower SS values for both Si and Ge FinFETs (∼40 mV/dec) possibly because of the use of a thin π-layer (3 nm) hence operating voltage (0.5 V). Typically, for n-type FETs, lower SS values are obtained which can be explained by the higher deformation potential in the conduction band than that in the valence band [42]. Moreover, Ge is the most attractive material because it has a lower Young’s modulus and higher deformation potentials compared to the other bulk materials.

**Figure 6:**
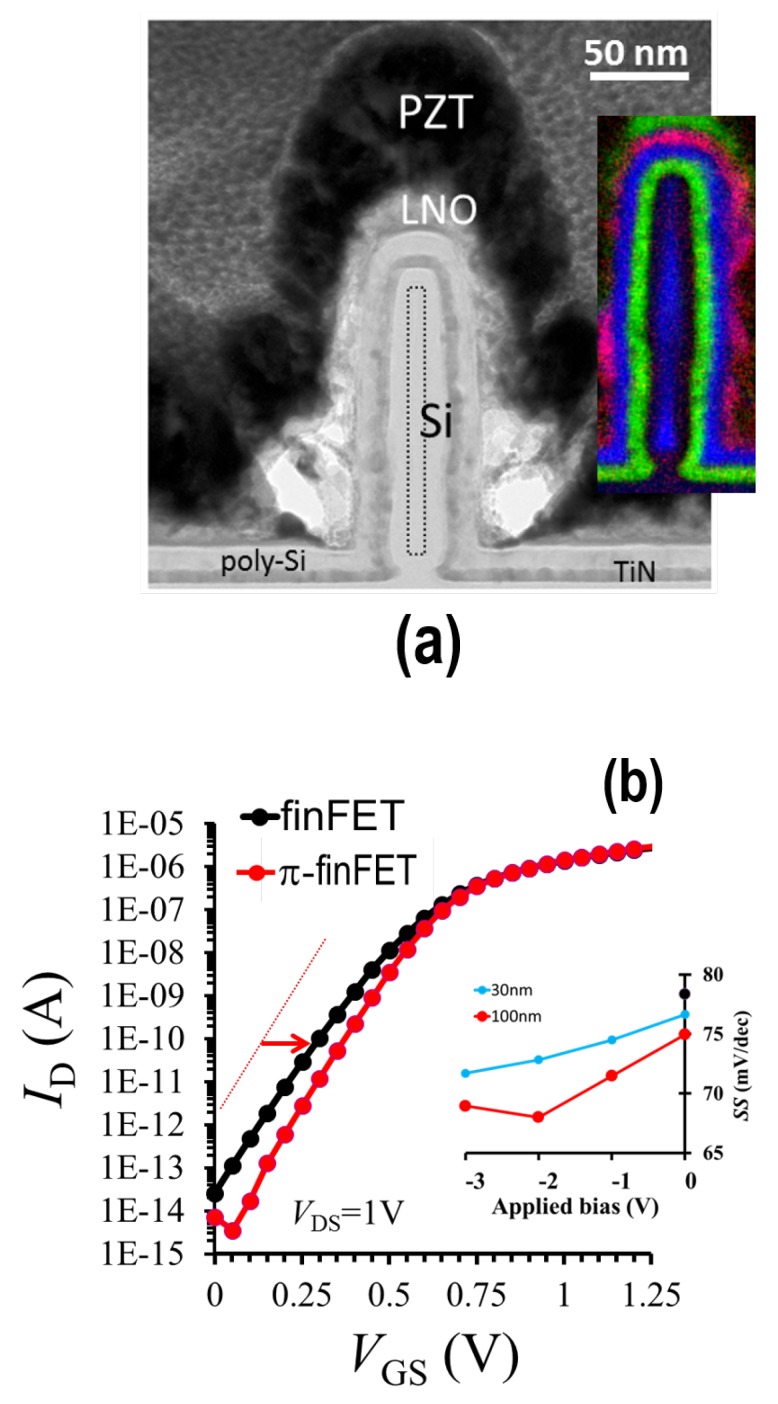
(**a**) High-resolution transmission electron microscopy (HR-TEM) image (incl. an red-green-blue (RGB) map: La is red, Ti is green, and Si is blue) of one fin of a silicon π-FinFET with a 20 nm fin width, and a 10 nm LNO (lanthanum-nickelate) and 100 nm PZT layer stack. The LNO layer is a buffer layer to avoid ferroelectric performance degradation of the PZT layer and atom interdiffusion through interfaces. The fin height is around 150 nm. In addition to the 10 nm LNO layer the gate stack comprises a 12 nm poly-Si, 10 nm SiO${}_{2}$ and 6 nm TiN (gate metal). (**b**) Current-voltage ($I_{D}$-$V_{\mathrm{GS}}$) characteristics of the π-FET compared to that of the conventional counterpart. Note that the π-FET curve has been shifted to match the $I_{\mathrm{ON}}$ ($\Delta V_{\mathrm{GS}}\approx$ 0.2 V). The inset shows the measured SS value against the (separate) bias over the π-layer for two different fin widths (30 and 100 nm).

For the first time, in collaboration with the company SolMateS B.V. (Enschede, The Netherlands), we had realized a prototype of the π-FET comprising an Si FinFET wrapped around by (buffered) PZT (see Figure 6) or AlN as a π-material [48]. That work resulted in several new insights. First of all, the buffered PZT layer around the FinFET did not degrade the Si, as confirmed by our electrical measurements and X-ray photoelectron spectroscopy (XPS) analysis. This indicates that a π-material with a high π-response such as PZT can in fact be integrated in Si active devices. Secondly, we observed a lower SS compared to that of the conventional Si FinFET counterpart despite the relatively thick, 11 nm, silicon-dioxide (SiO${}_{2}$) layer. Bulk SiO${}_{2}$ has a relatively low Young’s modulus (c${}_{11}$ = 57 GPa and c${}_{12}$ = 11.4 GPa) and consequently absorbs a big part of the strain [16]. Despite this fact, we observed that the converse π-effect reduced the SS by ∼5 mV/dec (but the SS was still higher than 60 mV/dec because of interface traps) which can be attributed to the strain-induced reduction of the trap density at the Si/SiO${}_{2}$ interface, as confirmed in the higher electron mobility values obtained in these structures at low vertical electric fields.

We also measured the π-response with the laser Doppler vibrometer [48], for the two π-materials (PZT: $d_{33}$ = 110∼110 pm/V, AlN: $d_{33}$ = ∼13 pm/V), indicating that the devices function well electromechanically. However, for an improved performance, the device should have been covered with a mechanically rigid layer to prevent mechanical energy losses in addition to the use of less, ultrathin and stiff interfacial layers. Furthermore, as discussed earlier [48], the conformality of the π-layer around the fin should be improved.

## 4. Discussion

In this section, we compare the performance for both device concepts based on previous reports. Then, we comment on some typical issues of those concepts.

As stated before, there have been numerous experimental papers on the NC-FET concept (e.g., [26,27,28,29,30,31,32,33,34,35]), all showing impressive results. In particular, the use of PZT in bulk MOSFETs [34] yield a record value in the SS, while, in FinFETs, equally impressive results were shown with HZO [32]. Numerical calculations on short channel NC-FinFETs predicted aggressive SS values [39] as well, though with artificial FE parameters. Unfortunately, an elaborate combined modeling and experimental effort on this device concept has been lacking so far.

For the π-FET, on the other hand, though much less has been reported compared to the NC-FET, less aggressive SS numbers are expected. This means that the NC-FET is superior from the viewpoint of static power consumption ($P_{\mathrm{stat}}$), and also $I_{\mathrm{ON}}$/$I_{\mathrm{OFF}}$ ratios since the $I_{\mathrm{ON}}$ has dramatically been increased by the elevated gate capacitance in addition to the reduction in threshold voltage. The expected increased channel mobility in the π-FET will result in an increased $I_{\mathrm{ON}}$ but less compared to that of the NC-FET.

For dynamic switching of the FE material, other parameters play a role [49,50,51,52]. In particular, because of the ∂P/∂t, the resistivity ρ (or damping constant, viscosity) of the FE material is important. This combined, with the fact that the NC-FET operation is based on a relatively high $C_{\pi }=\partial P$/∂E, ultimately show that the delay is determined by the total time constant. In perovskite FE materials, both parameters are relatively high [50,51], but still in hard FE materials, such as HZO, the switching times reach the MHz range [52] instead of the required GHz range for digital logic. Therefore, it was reported [50,51] that hard FE materials are required with lower ρ values. Alternatively, a relatively low $P_{r}$ could also help but that also depends on the transistor configuration. Note that, even without considering these dynamic FE effects, low switching speeds have been reported for the NC-FET using compact models [40].

For the π-FET, the switching speed is less of an issue since the device operates at relatively low ∂P/∂E values. The switching speed is basically determined by the sound velocity and the device dimensions [16,44]. Examples are manifold. There have been several reports on PZT bulk acoustic wave (BAW) resonators reaching GHz speed, for ∼1 μm thick layers [53,54]. This even applies to conventional piezoelectric (low polarization) AlN BAW resonators (e.g., [55]).

So far, the dynamic power consumption ($P_{\mathrm{dyn}}$) of both device structures have not been thoroughly investigated. For the NC-FET, some remarks have been given that the damping constant of the FE material governs the switching energy [50]. Moreover, several reports (e.g., [40]) where numerical calculations were performed indicate not a positive outlook (∼ tenfold increase in $P_{\mathrm{dyn}}$).

For the π-FET, it was estimated [42] that, depending on the device dimensions, the $P_{\mathrm{stat}}$ can drop by a factor of two in the silicon (Si) π-FinFET compared to a conventional FinFET. On the other hand, for the former mechanical power is needed during switching resulting in an increased $P_{\mathrm{dyn}}$, more or less within the same range. Based on prior International Technology Roadmap for Semiconductors (ITRS) roadmap estimations, this implies that, for less than ∼8 nm gate length, the total power consumption of the π-FinFET is less than that of the conventional FinFET. However, these are rough estimations and more accurate modeling and experiments are required to have better predictions.

In summary, amongst the discussed device structures, the NC-FET is most attractive for its relatively low $P_{\mathrm{stat}}$. The π-FET, on the other hand, appears to be more attractive from the viewpoint of switching speed and $P_{\mathrm{dyn}}$. Therefore, if no clear solution would have been found on tackling the speed issue of the NC-FET, a potential future low-power CMOS technology could be found in a hybrid solution comprising both device architectures on a chip where hard FE materials with a high $d_{33}$ are used. As indicated in Figure 1, the technology of both device concepts are not that different, which could imply that these are relatively simple to realize within a single production process. Table 2 summarizes the discussion.

For both device concepts, there are also specific requirements regarding the architecture. For the NC-FET, there should be sufficient charge in the close vicinity of the channel or body region. Fully depleted long channel NC-based FinFETs, nanowire (NW-)FETs, or perhaps even some two-dimensional (2D) material FETs, will mainly result in higher $I_{\mathrm{ON}}$ rather than lower SS. Bulk NC-FETs, on the other hand, will result in maximum performance (see, e.g., [34]). The NC-FET is sensitive to dimensional scaling because of this charge balance requirement. For the π-FET, this requirement is not important. For the π-FET, dimensional scaling is also relevant but more from a mechanical point of view and will be less sensitive to process variations. Conversely, the type of semiconductor in the body/channel strongly determines the performance of the π-FET because of its electro-mechanical properties, which is not the case for the NC-FET. For example, a theoretical study in which transition metal dichalcogenides (TMDs) as a channel material in a π-FET configuration was reported, referred to as the 2D-EFET [56], showing promising results.

Furthermore, the choice of FE material is also important. As stated before, for a good functioning of the π-FET, a high $P_{r}$ is required. For the NC-FET, on the other hand, this strongly depends on the device architecture, though a high $E_{C}$ is essential. Of course, materials such as HZO are more compatible in state-of-the-art CMOS technologies, but, for example, PZT can also be integrated in silicon technology, as adopted in Fe-RAM [18], or providing a proper buffer layer is used [48]. This discussion has been summarized in Table 3.

Obviously, there are many technological challenges, not only from the viewpoint of material science—especially when integrating FE layers in FinFET or NW-based FET technologies. First, the FE layer including the interfacial layers (e.g., gate metal, floating metal, buffer layer) should have a good conformality. In this way, a better gate control can be obtained throughout the device. Second, the conformal layers should be preferably of a minimal layer thickness so that edge or corner effects become less important. Third, for ultrathin FE layers (in particular for PZT [57]), a so-called dead layer is formed which causes a deterioration of the *P*-E loop, yielding lower $P_{r}$ values. A solution for some of those points may be to adopt atomic-layer deposition (ALD) in the process. ALD is an advanced deposition technique that is based on self-limiting surface reactions, yielding practically uniform, conformal layers. Furthermore, the whole gate stack can be formed without a vacuum break, which is important for interface control. Moreover, ALD is suited for an industrial setting. Note that the *P*-E loop can be further tuned for instance by adjusting the doping concentration (acceptor doping [Fe,La] for wider loops [19]) or by clamping (e.g., [20]).

Finally, some discussion about the temperature dependence for both types of devices is needed. Despite its importance, surprisingly, however, not much literature can be found regarding this issue. For instance, the work of Jo and Shin [58] report on the temperature dependence of the NC-effect. By connecting a ferroelectric capacitor to a conventional MOSFET, the authors experimentally showed an SS increase for elevated temperatures. On the other hand, in the milestone paper of Khan et al. [23], for instance, elevated temperatures were used (up to 500 ${}^{{}^{\circ}}$C, just near the Curie temperature of PZT) to show experimentally the NC-effect in a composite PZT/STO MIM capacitor. For the π-FET, so far there have been no reports in that direction.

Generally, it can be stated that the temperature has a strong effect on the properties of ferroelectric materials and consequently on the device performance for both types of devices. Therefore, more research is needed in that direction. Firstly, the so-called Curie temperature $T_{C}$, which is the minimum temperature at which a ferroelectric material becomes paraelectric (i.e., there is no hysteretic *P*-E curve), should be above the desired operating temperature range; otherwise, in principle, the devices won’t function properly. For both PZT (e.g., [23]) and HfO${}_{2}$ (e.g., [59]), it has been reported that this in the range of 500 ${}^{{}^{\circ}}$C, well above the operating temperature, but this will depend on the material composition and layer thicknesses. Even for high $T_{C}$, however, the polarization is sensitive to temperature variation. Second, ferroelectric materials are also so-called pyroelectric materials [17], which means that a surface charge in the ferroelectric material can be formed induced by a change in temperature.

## 5. Conclusions

The negative-capacitance field-effect transistor (NC-FET) requires capacitive tuning and is quite sensitive to any variation. For proper operation, a relatively wide polarization-field loop is required. The piezoelectric field-effect transistor (π-FET) requires a relatively high piezoelectric coefficient and hence the polarization-field loop, and special attention must be given to the mechanical boundary conditions that are important in this case. The NC-FET is by far superior in subthreshold-swing, on-current and static power consumption. However, literature data show that its dynamic properties (delay and power consumption) are inferior to those of the π-FET. Therefore, if the speed issue was not solved for the NC-FET, a potential future CMOS technology would be a hybrid solution comprising both NC-FET and π-FET in which hard ferroelectric materials with a relatively high piezoelectric coefficient have been incorporated.

## Figures and Tables

**Table 1:** Summary of analytically calculated SS values in π-FETs for various bulk semiconductor materials. nMOS and pMOS stands for an n-type and p-type MOSFET, respectively.

	SS (mV/dec), nMOS	SS (mV/dec), pMOS
Si	53.1	56.9
Ge	51.3	56.5
InSb	53.1	56.4

**Table 2:** A qualitative summary of the device performance of the NC-FET vs. π-FET.

	NC-FET	π-FET
SS	++	+
$I_{\mathrm{ON}}$	+/++	+
$P_{\mathrm{static}}$	++	+
$P_{\mathrm{dyn}}$	- -	-
τ	- -	+

**Table 3:** A summary of the device architecture requirements for the NC-FET and π-FET. * Charge required in the close vicinity of the body, ^§^ May be less effective.

	NC-FET	π-FET
Device type	Bulk/Fin*/NW*/2D*	Bulk^§^/Fin/NW/2D
Charge body	required	not important
Scaling	very important	important
Body/channel type (e.g., Si, Ge, TMD)	important	very important
Perovskite ferroics (e.g., PZT)	effective (record)	required
“Hard” ferroics (e.g., HfZrO${}_{2}$)	effective (concensus)	insufficient

## References

[1] Moore G.E. No Exponential is Forever: But “Forever” Can Be Delayed!. Proceedings of the International Solid-State Circuits Conference (ISSCC).

[2] Hisamoto D., Lee W., Keziekski J., Anderson E., Takeuchi H., Asano K., King T., Bokor J., Hu C. A Folded-channel MOSFET for Deep-sub-tenth Micron Era. Proceedings of the International Electron Device Meeting (IEDM).

[3] Saremi M., Afzali-Kusha A., Mohamadi S. (2012). Ground plane fin-shaped field effect transistor (GP-FinFET): A FinFET for low leakage power circuits. Microelectr. Eng..

[4] Colinge J.-P. (2008). FinFETs and Other Multi-Gate Transistors.

[5] Faynot O., Crisoloveanu S., Auberton-Hervé A.J., Raynaud C. (1995). Performance and Potential of Ultrathin Accumulation-Mode SIMOX MOSFET’s. EEE Trans. Electr. Dev..

[6] Welser J., Hoyt J.L., Gibbons J.F. (1994). Electron mobility enhancement in strained-Si n-type metal-oxide-semiconductor field-effect transistors. IEEE Electr. Dev. Lett..

[7] Chandrakasan A., Brodersen R. (1995). Minimizing power consumption in digital CMOS circuits. Proc. IEEE.

[8] Seabaugh A.C., Zhang Q. (2010). Low-voltage tunnel transistors for beyond CMOS logic. Proc. IEEE.

[9] Ionescu A.M., Riel H. (2011). Tunnel field-effect transistors as energy-efficient electronic switches. Nature.

[10] Imena Badi R.M., Saremi M., Vandenberghe W.G. (2017). A Novel PNPN-Like Z-Shaped Tunnel Field-Effect Transistor With Improved Ambipolar Behavior and RF Performance. IEEE Trans. Electr. Dev..

[11] Imena Badi R.M., Saremi M. (2018). A Resonant Tunneling Nanowire Field Effect Transistor with Physical Contractions: A Negative Differential Resistance Device for Low Power Very Large Scale Integration Applications. J. Electron. Mater..

[12] Gopalakrishnan K., Griffin P.B., Plummer J.D. I-MOS: A novel semiconductor device with a subthreshold slope lower than kT/q. Proceedings of the International Electron Device Meeting (IEDM).

[13] Salahuddin S., Datta S. (2008). Use of Negative Capacitance to Provide Voltage Amplification for Low Power Nanoscale Devices. Nano Lett..

[14] Salahuddin S., Datta S. Can the subthreshold swing in a classical FET be lowered below 60 mV/decade?. Proceedings of the International Electron Device Meeting (IEDM).

[15] Van Hemert T., Hueting R.J.E. Active Strain Modulation in Field Effect Devices. Proceedings of the European Solid-State Device Research Conference (ESSDERC).

[16] Van Hemert T., Hueting R.J.E. (2013). Piezoelectric Strain Modulation in FETs. IEEE Trans. Electr. Dev..

[17] Damjanovic D. (1998). Ferroelectric, dielectric and piezoelectric properties of ferroelectric thin films and ceramics. Rep. Prog. Phys..

[18] Setter N., Damjanovic D., Eng L., Fox G., Gevorgian S., Hong S., Kingon A., Kohlstedt H., Park N.Y., Stephenson G.B. (2006). Ferroelectric thin films: Review of materials, properties, and applications. J. Appl. Phys..

[19] Kimura M., Ando A., Sakabe Y., Uchino K. (2010). Lead zirconate titanate-based piezo-ceramics. Advanced Piezoelectric Materials. Science and Technology.

[20] Nguyen D.M., Dekkers J.M., Houwman E.P., Steenwelle R.J.A., Wang X., Wan X., Roelofs A., Schmitz-Kempen T., Rijnders A.J.H.M. (2011). Misfit strain dependence of ferroelectric and piezoelectric properties of clamped (001) epitaxial Pb(Zr_0.52_,Ti_0.48_)O_3_ thin films. Appl. Phys. Lett..

[21] Landau L.D., Khalatnikov I.M. (1954). On the anomalous absorption of sound near a second order phase transition point. Dokl. Akad. Nauk..

[22] Devonshire A.F. (1954). Theory of ferroelectrics. Adv. Phys..

[23] Khan A.I., Bhowmik D., Yu P., Kim S.J., Pan X., Ramesh R., Salahuddin S. (2011). Experimental evidence of ferroelectric negative capacitance in nanoscale heterostructures. Appl. Phys. Lett..

[24] Sze S.M., Ng K.K. (2007). Physics of Semiconductor Devices.

[25] Catalan G., Jiménez D., Gruverman A. (2015). Negative capacitance detected. Nat. Mater..

[26] Salvatore G.A., Bouvet D., Ionescu A.M. Demonstration of Subthreshold Swing Smaller Than 60 mV/decade in Fe-FET with P(VDF-TrFE)/SiO_2_ Gate Stack. Proceedings of the International Electron Device Meeting (IEDM).

[27] Rusu A., Salvatore G.A., Jiménez D., Ionescu A.M. Metal-ferroelectric-metal-oxide-semiconductor field effect transistor with sub-60 mV/decade subthreshold swing and internal voltage amplification. Proceedings of the International Electron Device Meeting (IEDM).

[28] Khan A.I., Yeung C.W., Hu C., Salahuddin S. Ferroelectric negative capacitance MOSFET: Capacitance tuning & antiferroelectric operation. Proceedings of the International Electron Device Meeting (IEDM).

[29] Lee M.H., Lin J.-C., Wei Y.-T., Chen C.-W., Tu W.-H., Zhuang H.-K., Tang M. Ferroelectric Negative Capacitance Hetero-Tunnel Field-Effect-Transistors with Internal Voltage Amplification. Proceedings of the International Electron Device Meeting (IEDM).

[30] Cheng C.H., Chin A. (2014). Low-Voltage Steep Turn-On pMOSFET Using Ferroelectric High-*k* Gate Dielectric. IEEE Electr. Dev. Lett..

[31] Dasgupta S., Rajashekar A., Majumdar K., Agrawal N., Razavieh A., Trolier-McKinstry S., Datta S. (2015). Sub-kT/q Switching in Strong Inversion in PbZr_0.52_Ti_0.48_O_3_ Gated Negative Capacitance FETs. IEEE J. Explor. Solid-State Comput. Devices Circuits.

[32] Li K.-S., Chen P.G., Lai T.-Y., Lin C.-H., Cheng C.-C., Chen C.-C., Wei Y.-J., Hou Y.-F., Liao M.-H., Lee M.-H. Sub-60 mV-Swing Negative-Capacitance FinFET without Hysteresis. Proceedings of the International Electron Device Meeting (IEDM).

[33] Khan A.I., Chatterjee K., Duarte J.P., Lu Z., Sachid A., Khandelwal S., Ramesh R., Hu C., Salahuddin S. (2016). Negative Capacitance in Short-Channel FinFETs Externally Connected to an Epitaxial Ferroelectric Capacitor. IEEE Electr. Dev. Lett..

[34] Park J.H., Joo S.K. (2016). Sub-kT/q subthreshold slope p-metal-oxide-semiconductor field-effect transistors with single-grained Pb(Zr,Ti)O_3_ featuring a highly reliable negative capacitance. Appl. Phys. Lett..

[35] Jana R.J., Snider G.L., Jena D. (2013). On the possibility of sub 60 mV/decade subthreshold switching in piezoelectric gate barrier transistors. Phys. Stat. Sol..

[36] Canon A., Jiménez D. (2010). Multidomain ferroelectricity as a limiting factor for voltage amplification in ferroelectric field-effect transistors. Appl. Phys. Lett..

[37] Salahuddin S. (2016). Personal Communication.

[38] Rollo T., Esseni D. (2018). Influence of Interface Traps on Ferroelectric NC-FETs. IEEE Electr. Dev. Lett..

[39] Hu C., Salahuddin S. 0.2 V Adiabatic NC-FinFET with 0.6 mA/μm *I*_ON_ and 0.1 nA/μm *I*_OFF_. Proceedings of the Berkeley Symp. Energy Efficient Electronic Systems & Steep Transistors Workshop (E3S).

[40] Pahwa G., Dutta T., Agarwal A., Chauhan Y.S. Designing energy efficient and hysteresis free negative capacitance FinFET with negative DIBL and 3.5X I_ON_ using compact modeling approach. Proceedings of the European Solid-State Device Research Conference (ESSDERC).

[41] Auld B.A. (1973). Acoustic Fields and Waves in Solids.

[42] Hueting R.J.E., van Hemert T., Kaleli B., Wolters R.A.M., Schmitz J. (2015). On Device Architectures, Subthreshold Swing, and Power Consumption of the Piezoelectric Field-Effect Transistor (*π*-FET). J. Electr. Dev. Soc..

[43] Starschich S., Schenk T., Schroeder U., Boettger U. (2017). Ferroelectric and piezoelectric properties of Hf1-xZrxO2 and pure ZrO2 films. Appl. Phys. Lett..

[44] Newns D., Elmegreen B., Liu X.H., Martyna G. (2012). A low-voltage high-speed electronic switch based on piezoelectric transduction. J. Appl. Phys..

[45] Synopsys, Inc. (2007). Sentaurus Device User Guide.

[46] COMSOL, Inc. (2011). Comsol MultiPhysics.

[47] Wang H., Jiang X., Xu N., Han G., Hao Y., Li S.-S., Esseni D. (2018). Revised Analysis of Design Options and Minimum Subthreshold Swing in Piezoelectric FinFETs. IEEE Electr. Dev. Lett..

[48] Kaleli B., Hueting R.J.E., Nguyen M., Wolters R.A.M. (2014). Integration of a Piezoelectric Layer on Si FinFETs for Tunable Strained Device Applications. IEEE Trans. Electr. Dev..

[49] Li J., Nagaraj B., Liang H., Cao W., Lee C.H., Ramesh R. (2004). Ultrafast polarization switching in thin-film ferroelectrics. Appl. Phys. Lett..

[50] Li Y., Yao K., Samudra G.S. (2016). Effect of Ferroelectric Damping on Dynamic Characteristics of Negative Capacitance Ferroelectric MOSFET. IEEE Trans. Electr. Dev..

[51] Yuan Z.C., Rizwan S., Wong M., Holland K., Anderson S., Hook T.B., Kienle D., Gadelrab S., Gudem P.S., Vaidyanathan M. (2016). Switching-Speed Limitations of Ferroelectric Negative-Capacitance FETs. IEEE Trans. Electr. Dev..

[52] Kobayashi M., Ueyama N., Jang K., Hiramoto T. Experimental Study on Polarization-Limited Operation Speed of Negative Capacitance FET with Ferroelectric HfO_2_. Proceedings of the 2016 IEEE International Electron Device Meeting (IEDM).

[53] Kirby P.B., Su Q.X., Komuro E., Zhang Q., Whatmore R.W. PZT thin film bulk acoustic wave resonators and filters. Proceedings of the 2001 IEEE International Frequncy Control Symposium and PDA Exhibition.

[54] Conde J., Muralt P. (2008). Characterization of Sol-Gel Pb(Zr_0.53_Ti_0.47_)O_3_ in Thin Film Bulk Acoustic Resonators. IEEE Trans. Ultrason. Ferroelectr. Freq. Cntrol..

[55] Van Hemert T., Reimann K., Hueting R.J.E. (2012). Extraction of second order piezoelectric parameters in bulk acoustic wave resonators. Appl. Phys. Lett..

[56] Das S. (2016). Two Dimensional Electrostrictive Field Effect Transistor (2D-EFET): A sub-60mV/decade Steep Slope Device with High ON current. Sci. Rep..

[57] Nguyen D.M. (2010). Ferroelectric and Piezoelectric Properties of Epitaxial PZT Films and Devices on Silicon. Ph.D. Thesis.

[58] Jo J., Shin C. (2015). Impact of temperature on negative capacitance field-effect transistor. Electr. Lett..

[59] Nishimura T., Xu L., Shibayana S., Yajima T., Misita S., Toriumi A. (2016). Ferroelectricity of nondoped thin HfO_2_ films in TiN/HfO_2_/TiN stacks. Jpn. J. Appl. Phys..

